# Not just political parties: Robert Michels as a critic of mainstream economics

**DOI:** 10.3389/fsoc.2023.1196264

**Published:** 2023-05-26

**Authors:** Emanuela Susca

**Affiliations:** Department Economia Società Politica (DESP), Università degli studi di Urbino “Carlo Bo”, Urbino, Italy

**Keywords:** Michels, Robert, economic reductionism, anti-economism, civil economics, sociology of consumption

## Abstract

Best known for his contribution to elite theory through the formulation of the principle of oligarchy, Robert Michels pursued a critique of economic reductionism for decades. In this paper, I examine some significant passages from Michels' writings to clarify the significance of his criticism of the dominant economics of his time. This provides an overview of an author partly conditioned by his adherence to Italian fascism but still able to distance himself progressively from productivist ideology while anticipating current lines of research focusing on the relationship between the market and society, such as civil economy. Moreover, by investigating how goods may provide happiness, Michels expressed a sophisticated and contemporary view of consumption, already bringing into focus the logic of distinction that Pierre Bourdieu examined in the second half of the twentieth century. By also attempting to do all this in an interdisciplinary way, Michels represents a scholar whom the social sciences and sociology should rediscover in the face of the challenges of the twenty-first century.

## 1. Introduction

As he lamented (see Trocini, 2020, p. 211), Robert Michels is best known for his studies of political parties or his idea that any organization is inevitably destined to evolve oligarchically. Moving beyond these aspects and reconnecting with some available investigations (Montesi, 2020, 2021a,b; Susca, 2020), the following instead deals with a different and particularly sociologically interesting aspect: the Michelsian view of the economy. In some passages of his writings, increasingly since the early postwar period and even more so since his adherence to Italian fascism, Michels distanced himself from classical and marginalist economics by proposing a stimulating vision of the social meaning of production and consumption that is close to contemporary sensibilities and useful for the problems and challenges that are arising today.

In summary, Michels, while holding professorships in political economy in Basel, Switzerland, and the Italian universities of Turin, Messina, and Perugia, expressed a position strongly opposed to economic reductionism. Moreover, he undertook a radical reformulation, intending to transform that same economics that, per Carlyle's definition, was a “dismal science” dealing with needs compressed by scarcity and overpopulation into a science of welfare and thus, to a large extent, of social justice or even human happiness.

In the first part, I show how Michels' aspiration took the form of a political and, more generally, cultural “third way,” an alternative to both Marxist and socialist materialism (which had also attracted Michels as a young man), as well as to Anglo-Saxon-derived liberalism. Moreover, I relate Michels' position to the ideological needs that arose during the consolidation of Benito Mussolini's regime. However, I also emphasize that Michels' view was not only an effect of his closeness to fascist ideology but also a consequence of his rediscovery of and appreciation for some ancient figures in Italian economic thought who had conceived of economics as inherently moral and social, thus representing a significant, though little known and valued, antecedent of the many currents and schools that today call for a decisive break from the idea of human beings that liberalism and neoliberalism express. Seen from this angle, the arguments and insights emerging from Michels' work prove insightful and progressive in their ability to anticipate future directions in the social sciences and, above all, to provide tools and keys that are more useful today than ever before.

Next, I turn to Michels' critique of the maximizing rationality and abstraction of *homo oeconomicus*. In doing so, I highlight how this *sui generis* scholar advocated a realistic view of action, conceptualizing in relational and, in some ways, communitarian terms both the properly human activity of work and the equally human pursuit of wellbeing (and a wellbeing that is not reduced to the economic aspect alone).

In the last section, I briefly analyze the explanation of consumption that emerges from examining some of Michels' observations. Specifically, I attempt to show how he tended to omit or disregard as central some of the motivations for consumption that were and often are considered fundamental, such as the mere gratification of needs, conspicuous ostentation, or even quasi-mechanical imitation. Rather, through his fully social and relational vision mentioned above, he objectively anticipated a perspective centered on the “distinction” between social classes that Pierre Bourdieu would formulate more clearly only decades later.

Finally, the concluding remarks point out how Michels was able to preconceive and bring into focus opaque areas and aporias of common ideas about economic growth and the pursuit of profit while also demonstrating a remarkably intellectual open-mindedness in crossing those disciplinary boundaries that today appear outdated or at least to be deeply rethought.

Since Michels is considered a classic of sociology but his refutation of economic reductionism has been ignored by sociologists and analyzed from an economic perspective, it is appropriate to examine this specific aspect sociologically in order to bring forward a reconceptualization of social behavior and action that is more necessary than ever.

## 2. Convergence with fascism and economic anti-reductionism

To understand Michels' position with respect to traditionally understood economic science, a passage from *Il coefficiente psicologico dell'economia politica* (*The Psychological Coefficient of Political Economy*) can be recalled. Here, Michels wrote:

The concept of the economic man may be useful for the precise purposes of pure economic research. However, its usefulness is strictly limited to abstraction. Applied to economic life, the theory of economic man threatens to mutate from abstraction to mere liberaloid fiction, capable only of diverting and altering the real course of things, as well as creating, in the name of alleged scientific truths, unjustified impediments to the action of the state and of generous, energetic, and progressive individuals (Michels, 2001, p. 37).

The publication from which the above quotation is taken is from 1928, a period by which this author's youthful closeness to socialism had long ended. Michels changed his political views mainly because he was deeply disappointed both with the cautious reformism of many European socialist and social democratic leaders and with Marxism itself. In the latter particularly, he now saw an economicist philosophy of man and society, something too materialist to recognize the crucial role that cultural aspects and ideals play (Michels, 1949, p. 24–62). Even after abandoning the socialist movement and deepening his knowledge of neoclassical and marginalist economic science, Michels did not, however, become a liberalist. Contrarily, he moved closer to fascist ideology and basically shared the position on economics prevailing in Benito Mussolini's inner circle of men. Indeed, Italian fascism oscillated for a few years between liberalist and nationalist positions (Michelini, 2019) and then switched to a basically nationalist and corporatist idea of the economy after a brief period.

In this light, the political significance of Michels' criticism of *homo oeconomicus* is clear. This term, besides being an abstraction of doubtful utility for investigating concrete economic phenomena, appeared to Michels as the essentially Anglo-Saxon product of a dangerous “liberaloid” drift. For him, therefore, that essentially individualistic and selfish view of the economic subject had to be countered to prevent any impediment to state action and, especially, to allow Mussolini's political genius to unfold freely.

Michels was suggesting to fascism what fascism itself was pursuing in those years, that is, a type of third-way alternative to both materialist socialism and the English-derived economic orthodoxy radicalized by marginalism. This is well-evidenced in the aforementioned *Il coefficiente psicologico dell'economia politica*, according to which the fascist regime is thought to be even more decisively “voluntarist” and “statist,” even in economic choices, thus preventing any supposed economic law or particularistic instance to impede the Duce's leadership and the organic union of the political community (Michels, 2001, p. 53).

However, Michels' position was not only immediately political, nor can it be interpreted solely as a demonstration of ideological support or adherence to fascism. In fact, in addition to dubious or more elementarily mistaken political choices, he took a stance against economic reductionism, which had matured through the in-depth study of Italian authors who had long since rejected the idea of economics as a science far removed from morality and devoid of responsibility toward society. Two figures almost forgotten today can be recalled in this regard: Lodovico Antonio Muratori, who wrote a treatise entitled *Della pubblica felicità* (*About Public Happiness*), which affirms that happiness itself flows from civic virtues and not from the pursuit of individual self-interest; Giuseppe Pecchio, who advocated an Italian economic science manifesting “amor patrio” (love for the homeland) well before the political unification of Italy and with whom Michels himself ideally conversed (Michels, 2011, p. 32; see Mornati, 2012). Hence, born German and becoming Italian by choice, Michels chose to make himself heir to that tradition of economic thought, thus objectively becoming an anticipator of that tradition's current outcomes.

## 3. The project of refounding economics

As has been rightly observed (Montesi, 2020, p. 97–101), there are several similarities and points of contact between Michels and the “civil economy,” a significant economic current that has as some of its main exponents the Italians Stefano Zamagni and Luigino Bruni and that criticizes the dominant economy based on the alternative ideas traceable to humanism and the age of Enlightenment. The intent of these socially sensitive economists is to seek a reconciliation between, on the one hand, the market, long and catastrophically considered indifferent to anything but exchange or profit, and, on the other hand, society and human sociality as they are more broadly understood. Additionally, the subject, in addition to obviously producing and buying, is and remains a social being and is, therefore, moved by motivations that extend beyond self-interest and should also be understood in relation to the expressive and symbolic planes (Zamagni, 2005, p. 156–158). Moreover, these economists decisively distance themselves from *homo oeconomicus* by recognizing in that abstraction the “social idiot” type, that is, a model of an actor who is self-referential and so selfish that they do not realize that even their own economic profit is inextricably connected to that of others (Bruni and Zamagni, 2007, p. 149).

However, there have been numerous directions in economic studies that insist on the need to rethink both the market economy and the economic actor in different terms in recent decades (Susca, 2019). Here, Michels was an anticipator of these new directions and views to the extent that he wanted to resume the eudemonistic purpose that economics had gradually lost in its own classical and then neoclassical and marginalist formulation. To do so, in as early as 1918, he wrote *Economia e felicità* (*Economics and Happiness*), a work with an emblematic title that stemmed from the effort to put the great theme of human happiness back at the center of economic analysis. In the introduction, which appears as a programmatic manifesto, Michels wrote:

Man's deepest and most intense desire is to achieve happiness. Yet happiness constitutes the supreme end of all human institutions, the purpose of all orders and systems, state and religious. Economics also tends to this end, to which it is subordinated as a *medium ad finem*. Thus, economics cannot, as some have believed, consist in the search for and doctrine of the means to increase production, but rather it is appropriate for it to deal with production only insofar as it is likely to increase for men the possibility of living contented lives; that is, with production in its relations to the distribution and consumption of consumable products (Michels, 2017, p. 25).

When one considers to what extent the criticism of productivist ideology and the thematization of a broader idea of wellbeing have now become central topics, the considerations just mentioned seem extremely similar to the growing sensibility in our time. Moreover, Michels focused his inquiry on the idea of progress, which is the implicit presupposition of the view that conceives economic growth as a dogma. Indeed, in his 1919 essay devoted to progress, he did not merely express his appreciative sympathy for the masses once impoverished by the rise of capitalism or for the harsh living conditions that still afflicted most workers (Michels, 2011, p. 47 ff.); stressing that “the fundamental character of progress is that it is never complete” and that the “nature” of progress is “fragmentary, contradictory, occasional” (Michels, 2011, p. 53), he called attention to two truths that are difficult to refute but are too often forgotten. First, there is a real possibility that some people must be worse off for others to be better off. Second, technical or economic progress may not coincide with social progress; it may be the cause of the worsening of many people's lives.

In this way, it is also possible to better grasp the significance of the rejection of *homo oeconomicus* to which I have alluded. When he contrasted the image of a selfish and asocial actor with the idea of a subject tied to its surroundings, Michels showed his political motivational drive: his adherence to fascism. However, he was also moved by the idea, matured over the years, that the economy should be deconstructed in a communitarian sense to bring the focus back to the needs of society.

Moreover, he was moved by epistemological and methodological concerns that were crucial and that related to the reflections of one of the most important personalities who had most influenced his theories, Vilfredo Pareto, the great sociologist whom some consider the father and systematizer of *homo oeconomicus* (Demeulenaere, 1996, p. 157) but who was conscious of how that concept could be at best a useful abstraction for economists (Pareto, 1897, par. 1086–1087; Pareto, 1971, chap. 9). As an excellent disciple of Pareto, Michels had a view of *homo oeconomicus* devoid of any hypostatization and that was purely instrumental. However, he was also aware, perhaps more so than Pareto himself, of how problematic it is to analyze economic aspects by isolating them from supposedly non-economic ones. He emphasized this in his *Corso di sociologia politica* (*Course in Political Sociology*), in which he stated that “the economic way of life rarely admits distinct and clear separations from the other ways of human life” (Michels, 1949, p. 24) and that “in real life, the absolute abstraction of the economic man who is ruled over by economic principles does not exist” (Michels, 1949, p. 27). The epistemological value of this is emphasized in *Il coefficiente psicologico dell'economia politica*, in which Michels stated that “one cannot easily separate, even for scientific purposes, the zones in which pure economic man moves” (Michels, 2001, p. 60). Nor did he fail to draw conclusions from his own reasoning. As he made clear in one of his writings a few years prior to 1936, the year of his death, he became convinced that “the unreal concept of homo oeconomicus” had served and could serve at best an artificially abstract science so incapable of interpreting reality that it dismissed as merely “disturbing” all phenomena and behavior that contradicted it. In his opinion, therefore, that one-sided science had to be abandoned in favor of a vision open to the wholeness of concrete life and the specific sciences that investigate the manifold aspects of that life (Michels, 1932, p. 60).

In summary, Michels' viewpoint does not appear to have been motivated solely by considerations classifiable as political (fascist statism) or moral (condemnation of capitalist productivism). In fact, his position was also epistemological, giving rise to a program refined over years (or rather decades) and that—progressively anticipating contemporary research directions, as mentioned—was aimed at the refounding and relocation of economics. Therefore, despite claims to the contrary (see Gregor, 2015, p. 36), Michels was not a confusing scholar with little knowledge of economics. Although he knew economics in general and pure economics especially quite well, he intended to argue for a profoundly different orientation from the prevailing one.

With a conception similar to that of civil economics, as I have discussed, Michels intended to bring political economy back into the realm in which, in his view, it belonged, namely that of the “moral sciences,” and, above all, to help make political economy a perspective “aware of the infinite reciprocity of causes and effects which distinguishes all human activity” (Michels, 2001, p. 61). He went so far against the mainstream position of his time that he stated that “there is no place in science” for pure economics (Michels, 1934a, p. 17–18). Moreover, he supported the idea that “the very mechanism of political economy is totally interpenetrated by psychological categories” (Michels, 2001, p. 26), deriving from this assumption the project of breaking down the boundary between economics and psychology, something to which I will return briefly in my concluding remarks. In this sense, too, he was a learner of Pareto who intended to go beyond the reflections of his master. Just like Pareto, a theorist of the “residual” and non-logical, Michels refocused the sides of social life and behavior to a point irreducible to the model of maximizing rationality. However, viewed from the multidisciplinary or transdisciplinary perspective, the Paretian scientific project can be summarized into two assumptions. First, the intersection between psychology and economics is entrusted to economic psychology, which, like any other branch of psychological studies, must, for Pareto, measure itself against observable “facts,” thus proceeding inductively and not deducing anything from the deeper psychic dimension (Pareto, 1935, par. 2078 note). Second, Pareto considered the relationship between economics and sociology as an encompassment of the former by the latter, with sociology thus becoming so “general” as to embrace the “results” and ultimately the very object of pure economics and economics in general (Pareto, 1935, par. 2013; see Susca, 2005, p. 14–23). Aiming rather at hybridization, Michels intended to contribute to the construction of a new boundary science that would be both economic and sociological, supported moreover by the categories and acquisitions of psychology.

## 4. Explaining consumption sociologically

Overall, commentators have paid little attention to the fact that Michels, by bringing sociology and economics into dialogue, offered interesting and even anticipatory insights into the complexities of human behavior thanks to his viewpoint of consumption. In keeping with his ambition to counter economicism, he opposed the marginalist and generally liberalist viewpoint that still almost exclusively emphasizes the satisfaction of needs and the unquestionable choices of subjects. Mainstream economics tended and tends to refuse to address what is consumed and why it is consumed, so Michels applied to this matter his own idea of the social and economic actor distanced from the abstraction of the solitary individual and “absolute sovereign.” Consequently, though not organically and mainly through synthetic observations, he conceptualized and represented consumption as a social and relational phenomenon (or complex of phenomena), thus conceiving of the symbolic function of what is purchased and the capacity of goods as the creation of relationships among people (and not just the gratification of selfish desires).

In this regard, from the end of the nineteenth century onward, sociology began to experience a general movement of interest in an aspect that economics neglected and undervalued, that is, the sphere of consumption at that time. Similar to this is the conspicuous consumption that Veblen (1899) theorized or Simmel's (1895) sharp reflections on fashion. Therefore, it is unsurprising that Michels turned his analysis to consumption, interpreting it as a vital aspect in social life.

This aspect is evident in the considerations I referred to above drawn from the introduction to *Economia e felicità*, in which it is stressed how important it is to deal with production while always considering that the purpose of production is to increase “the possibility of living contented lives” by enabling people's “consumption of consumable products” (Michels, 2017, p. 25) on a large scale. However, Michels had a sophisticated idea of the nexus between consumption and happiness. Regarding the latter, he did not understand contentment abstractly or mechanistically; he instead thought of changing conditions as explainable only in relation to society and social changes. This is evident in some interesting remarks about “the increase in real wages” producing, in addition to an increase in available “wealth,” an increase in “social needs.” Indeed, Michels claimed that economic-type development based on an increase in spending capacity is “devoid of progress.” He argued this as follows:

Marx well said that “needs and enjoyments arise in society and are measured in society” and that “we do not measure them in the objects that serve their satisfaction, but that, being social in nature, they are relative in nature.” The concept of poverty and the concept of wealth should also be understood in this sense. [...] For every progress made creates a new need and, along with it, the desire to have a means that is believed to end it by satisfying it. In short, it could be said that apart from absolutely basic needs, need is merely the psychic repercussion of a given economic environment and civilization (Michels, 2001, p. 145–146).

Michels cited Marx in support of his own viewpoint, evidently seeing in the latter an illustrious precedent of his own attempt to refute economic reductionism by valuing the interference of psychology in economics. However, one can juxtapose his argument with the considerations that Simmel had already developed at the time, that is, those made by an author whom Michels himself appreciated and who can still be considered a classic point of reference on relative poverty because of his ability to grasp indigence relationally, in addition to his attention to modernity and urban life.

Regardless, Michels also dealt with envy from a sociological viewpoint. In fact, in the aforementioned essay, *Economia e felicità*, he highlighted that the various social classes, and particularly the working classes, make a comparison whenever they assess their own wellbeing. According to him, this means that “the economic improvement of one class does not penetrate into the *consciousness* of that class except when the improvement is at least proportional to that of the other classes” (Michels, 2017, p. 159). Accordingly, he spoke of a “misery of comparison” that affects people like a “disease,” even people who “enjoy undeniable economic-social improvement” but fail to rejoice in it, wisely observing that “comparison kills, by its very nature, budding happiness” (Michels, 2001, p. 162). Nor did he forget to consider the upper classes, to which he addressed some observations:

The rich man can sublimate his economic needs by assuming the character of a patron, social benefactor or collector, and derive new forms of satisfaction from them. If, however, he does not have the aptitudes and dispositions to achieve this sublimation, and if he is reduced to moving instead on the actual economic terrain, the arch-millionaire is unable to make his millions work in the direction of a continuous increase in the *chance* of happiness. Happiness does not go hand in hand with increased prosperity (Michels, 2001, p. 169).

The above considerations go beyond trivially noting that money does not lead to happiness or that the envy of the less-favored classes is often matched by the boredom of the richer ones. Rather, as the mention of the case of the “collector” shows, Michels meant to say that wealthy or extremely wealthy people can escape from the frustration that affects those who have no one to rival them by increasing the symbolic value of at least some of their possessions at the expense of their more common and immediate use value. Furthermore, Michels also proved to be insightful and progressive in the example of the “social benefactor.” Indeed, in times like ours, when a new type of philanthropy has become so widespread among mega-corporate tycoons as to lead some to speak of philanthrocapitalism (Bishop and Green, 2008; Dentico, 2020; McGoey, 2021), charity is arguably not only a way for millionaires to increase their influence or alleviate their taxes but also a means of meeting some psychic need.

However, Michels' sociological perspective on consumption is also relevant because, although not expressed systematically, it already represents an attempt to grasp the aspect of “distinction” between social classes that Bourdieu (1984) would highlight only decades later. This is particularly evident in *Il coefficiente psicologico*, in which Michels was not only referring to the imitative dynamic that Tarde theorized or the conspicuous consumption that Veblen analyzed, nor even to envy as a universal psychic motivator. Speaking specifically of fashion, in addition to recalling Simmel's suggestive studies on the subject, he spoke explicitly of a “purpose of distinction,” observing that fashion “has no other end than that of preventing the lower classes from erasing the barriers” that “separate them from the higher classes” (Michels, 2001, p. 41). He also drew a more general consideration from that example, as he affirmed the existence of a “human tendency to distinguish oneself” through which consumers choose goods and adopt consumption styles to emphasize their distance from those in an inferior condition (Michels, 2001, p. 48). Moreover, reasoning specifically about fares for train travel, Michels pointed out that the reason for the difference in cost among categories was ultimately not the “different degree of comfort and convenience” for travelers but “the shrewdness with which the railway administration exploits the public's desire to classify itself” to itself and others. In fact, he observed that there are people who travel first class to show that they can afford it (i.e., according to a model of ostentatious consumption) and people who do the same thing to give the impression that they can afford it, i.e., “*modest* people who have an interest in *appearing* to *become*” (Michels, 2001, p. 46), thus behaving according to the model now called “anticipatory socialization.”

In summary, there is a viewpoint that considers the power relations expressed by consumption by conceiving of the consumer acting according to distinctive and socially directed logic. In this regard, it can be argued without exaggeration that Michels may have anticipated, but also influenced, a contemporary sociologist as famous today as Bourdieu. Although he quoted little from Michels, as well as a number of other sources from which he drew inspiration (Riley, 2017, p. 127), Bourdieu knew in detail at least some of Michels' work (Bourdieu, 1991, p. 277, 283–285). It is true that the French sociologist intended his critique of domination as a contribution to overcoming or at least weakening domination itself, thus inevitably distancing himself from the elitist principle that the popular masses are by their nature destined to be and remain subjugated. However, the points of contact with Michels' perspective, namely a not insignificant affinity (Lapeyronnie, 2006, p. 13), also extend to the sociological view of culture and consumption in particular. The considerations just seen about being a first-class passenger should therefore not be read only or so much as a conceptualization of conspicuous consumption enacted by the rich who have to prove that they can afford everything and enjoy luxury. Rather, these considerations bring into focus the consumption choices made by those who sacrifice themselves by spending more than necessary to travel, so as not to sit next to workers and more modest people, that is, by the petty bourgeoisie that enters “the game of distinction” anxious to expose themselves to classification, which we find portrayed years and years later by Bourdieu (1984, p. 57).

## 5. Conclusions

Known primarily for his principle of oligarchy and contribution to elitist theory, Michels was and is often hastily labeled exclusively as a sociologist of politics, remaining arguably overshadowed by the fame of two renowned “neo-Machiavellians,” Vilfredo Pareto and Gaetano Mosca (Burnham, 1943). However, there are other lesser-known aspects of Michels, such as his capability for real “prophetic daring” (Federici, 2011, p. 8). Among the latter is the penetrating criticism of the dominant economics of Michels' time, as well as of our time, and the consequent adoption of an alternative perspective attentive to broader and more concrete wellbeing. This is a great legacy considering that, for the social sciences in general and for sociology in particular, it is more appropriate than ever to value the theoretical insights and research that have gradually challenged and criticized the economic dogmas that have problematic or even disruptive outcomes today.

Further, Michels was an inspiring author because of the lack of prejudice and open-mindedness with which he placed himself “at the frontier,” eschewing the divisions among the sciences and instead attempting to revive or offer his own contribution to a “frontier science” enriched by the dialogue among different scientific perspectives (Gioia, 1989, p. 55–56). Additionally, this compelling interdisciplinary approach was explicitly stated in all of his works, even in those that appear to be works of political sociology. Indeed, even in presenting his own analysis of the party form, Michels positioned that work of his in “an intermediate field between the social, the philosophico-psychological, and the historical disciplines” (Michels, 1915, p. VIII). In this way, he was already manifesting a vision similar to that expressed shortly before his death, calling for “overcoming the abstraction of economics” or grasping “the connections” of economics “with other points of view” while pursuing a “direct relationship with politics and philosophy” (Michels, 1934b, p. IX).

In addition to his adherence to fascism, Michels could be considered to have shown eclecticism in pursuing his ambitious scientific agenda. However, he displayed an inexhaustible curiosity and an impressively receptive mentality, being and remaining a hard-to-label figure. Wanting, however, to categorize him, he can also be considered a scholar of economic sociology or sociological economics (Faucci, 1989, p. 38–39) who occasionally applied a transdisciplinary viewpoint to a range of research objects: parties, industrialism, misery, national and ethnic differences, and sexual and gender differences. While there is sometimes a gap between the goals that he stated and the results that he achieved, Michels died rather suddenly at the age of 60, so what he would have done or written had he lived longer is unknown. In all likelihood, he would not have distanced himself from Italian fascism, thus casting further doubt on his political realism and elitism. However, looking at less immediately political issues, he may have measured himself more thoroughly and organically with some of the intellectual challenges he already glimpsed and which arise even in an age such as ours, in which the economy still dominates too often.

## Author contributions

The author confirms being the sole contributor of this work and has approved it for publication.
